# Effects of the Omicron variant on perinatal outcomes in full-term neonates

**DOI:** 10.1186/s12887-022-03690-8

**Published:** 2022-11-03

**Authors:** Hyowon Choi, Eun Jung Lee, Yeon-Soon Ahn, Yeong Myong Yoo

**Affiliations:** 1 Department of Pediatrics, Wonju Severance Christian Hospital, Yonsei University Wonju College of Medicine, 20 Ilsan-Ro, Wonju, Kangwon-Do 24626 South Korea; 2 Department of Prevention Medicine, Yonsei University Wonju College of Medicine, Wonju, South Korea

**Keywords:** COVID-19, Perinatal outcomes, Full-term neonate, Omicron variant

## Abstract

**Background:**

Research of coronavirus disease (COVID-19) effects on newborns is ongoing. But the research of specific variant’s effects is none. This study analyzed the effects of the Omicron variant on the perinatal outcomes of full-term newborns during the Omicron wave period.

**Methods:**

Between December 2021 and April 2022, this study was conducted on all newborns who visited a single center. We investigated due to the Omicron maternal infection maternal pregnancy complications, delivery methods, birth week, Apgar scores, neonatal resuscitation program requirement, whether respiratory support was required until 12 h after childbirth, suspicious infectious status, and mortality depending on maternal Omicron infection.

**Results:**

A total of 127 neonates were enrolled, and 12 were excluded based on exclusion criteria. Sixteen neonates were born to mothers with a history of Omicron COVID-19, and 99 were born to non-infectious mothers. All infected mothers became infected in the 3rd trimester. Of the 16 mothers, seven were symptomatic, and four met the isolation criteria, according to Korean guidelines. The birth weight of newborns to mothers with a history of COVID and those without was 2.958 ± 0.272 kg and 3.064 ± 0.461 kg (*p* = 0.049), respectively. The 5-min Apgar score at childbirth was 9.29 ± 0.756 and 9.78 ± 0.460 for neonates born to symptomatic and asymptomatic mothers (*p* = 0.019), respectively. When compared with or without maternal self-isolation, neonates requiring respiratory support 12 h after birth demonstrated a significant difference (*p* = 0.014; OR, 10.275). Additionally, the presence or absence of transient tachypnea of the newborn showed a significant value (*p* = 0.010; OR 11.929).

**Conclusions:**

Owing to Omicron COVID-19, newborns were born with lower birth weight, low 5-min Apgar scores, and required respiratory support until 12 h after birth.

## Background

The coronavirus disease 2019 (COVID-19) is an infectious disease, identified in December 2019, and has had a major global impact; various mutations of COVID-19 have developed since then [1]. Moreover, various studies have been conducted on COVID-19, but its effects on humans remain unknown [2]. In addition, recent studies reported that the impact of COVID-19 on immunologically vulnerable populations may be more dangerous than that in other populations [3]. Further, research on the effects of COVID-19 on newborns is ongoing, and it has been reported that cases of direct infection of newborns born to infected mothers are rare. Other than meta-analyses, few studies have analyzed the additional complications that may occur in the affected newborns. Additionally, the reported meta-analyses are based on a case series. However, in the previous studies, there were no differences in preterm birth and neurological outcomes in the neonates born to mothers with COVID-19 and uninfected mothers [4–10]. Furthermore, very few studies have been conducted on full-term infants [11].

In addition, it is known that the novel coronavirus (2019-nCoV) continues to mutate and that there are various mutants, each exhibiting differences in infectivity, virulence, and antigenicity. Therefore, numerous studies are being conducted for each known mutation [1]. However, there have been few studies on how each variant affects newborns, and fewer studies on respiratory symptoms that can occur in newborns [4–10].

Currently, in Korea, the proportion of mothers receiving antenatal care is high, and childbirth mostly occurs in hospitals [12]. Therefore, as COVID-19 spreads, all mothers are undergoing screening tests before or after visiting the hospital for infection control. In addition, since December 2021, Omicron has become the dominant variant, and self-test kits are used clinically [13]. If a mother tested positive for COVID-19 through antigen–antibody or polymerase chain reaction (PCR) tests, 2019-nCoV was confirmed. Thereafter, the newborns were isolated for 48 h and released from quarantine following a negative test result, based on the Korean Society of Pediatric Infectious Diseases guidelines. Additionally, in the case of the previous infection, if the cycle threshold (Ct) values of the RNA-dependent RNA polymerase (RdRp) gene or envelope (E) gene were 25 or less through PCR testing, it was considered a re-infection, and the newborn was quarantined [14]. Therefore, most patients in Korea are admitted to the neonatal intensive care unit.

This study investigated the effects of Omicron COVID-19 infection on general and respiratory outcomes of full-term infants hospitalized for isolation owing to infected mothers, compared to babies who had never been infected.

## Methods

Between December 1, 2021, and April 30, 2022, a retrospective study was conducted on all newborns aged 37 weeks or older who were admitted to the neonatal unit of Wonju Severance Christian Hospital. Newborns who were hospitalized more than 3 d after birth or who had a congenital anomaly were excluded from the study. All mothers of newborns were tested for 2019 nCoV before delivery or within 24 h of hospitalization, using PCR (SD Biosensor, Inc. STANDARD™ M10 SARS-CoV-2, South Korea, SD Biosensor, STANDARD™ M nCoV Real-Time Detection kit, South Korea). Mothers who tested negative on PCR were screened for COVID-19-related symptoms before admission Furthermore, patients with an unclear history of infection and mothers infected during wave periods of other variants were excluded from the study.

The management of pregnant women with COVID-19 was based on the latest Korean Society of Pediatric Infectious Diseases guidelines [14]. Mothers who had symptoms of upper respiratory tract infection such as cough, runny nose, or phlegm, or who had previously tested positive for 2019-nCoV on PCR, gave birth in isolation within 7 days while wearing protective equipment. Further, those with past infections were isolated as per the above protocol if the Ct values of RdRp and E genes were 25 or less. Their infants were isolated in the neonatal intensive care unit negative pressure room and tested for 2019-nCoV using nasopharyngeal PCR swabs at 24 and 48 h after birth. Newborns testing positive on any test were isolated for 7 d and released without test. If vital signs were unstable, hospitalization was maintained even after isolation was lifted. Based on the Korean Disease Control and Prevention Agency guidelines and the results of 2019-nCoV tests, mothers were divided into those with a positive confirmed history, those with COVID-19 symptoms, and those self-isolating for 7 d [13].

All mothers were investigated for age and complications during pregnancy, such as gestational hypertension, pre-eclampsia (G-HTN), prolonged rupture of membranes (PROM), and gestational diabetes mellitus (GDM). Based on the delivery method, the analysis was divided into vaginal delivery (VD) and cesarean section (CS). In addition, multiple fetuses, intrauterine pregnancy weeks (IUP), birth weight, Apgar score (A/S), and neonatal resuscitation program (NRP) requirements were investigated. Further, neonates were classified into small-for-gestational-age (SGA), average-for-gestational-age (AGA), and large-for-gestational-age (LGA) according to the weight by number of weeks [15]. For all newborns, we analyzed whether respiratory support was required until 12 h after birth, what the cause of respiratory compromise was, whether there was a case of suspicious sepsis, and mortality.

Transient tachypnea of the newborn (TTN) was defined when respiratory support was required until 12 h after birth and a typical sunburst pattern was seen on the infantogram. Respiratory distress syndrome of newborn (RDS) was defined as the presence of ground grass opacities or air-bronchograms on an infantogram, and a required FiO_2_ > 40%. In all RDS cases, surfactant inhalation was treated with intubation. Air leak was also defined as the presence of pneumomediastinum, pneumothorax, or pneumopericardium on an infantogram.

The analysis was conducted based on the mother's confirmed COVID-19 history, symptoms, and whether quarantine was enforced. Maternal age and birth weight were analyzed using a t-test, IUP (weeks), and A/S according to the Mann–Whitney U test. Chi-square verification was used to analyze the birth method, multiple fetuses, presence of oxygen at birth, presence of respiratory support until 12 h after birth and its cause, suspicious sepsis, and mortality. However, when the number was 5 or less, statistical analysis was performed using Fischer's exact test. All test values were analyzed using the SPSS 26.0 version (IBM SPSS Statistic, USA). Statistical significance was based on *p* < 0.05.

## Results

**Fig. 1:**
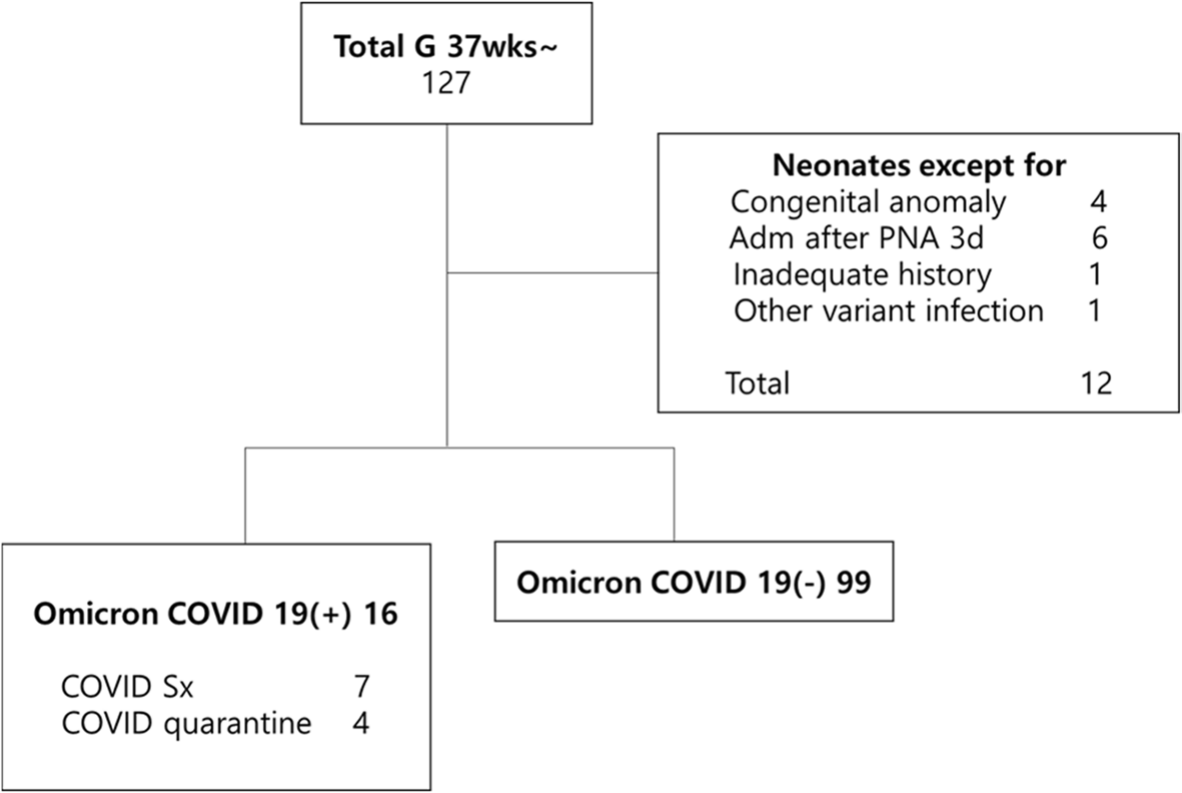
Population identification

A total of 127 full-term newborns were enrolled in this study. Six were excluded because they presented to the hospital 3 d after birth, and four were excluded owing to congenital anomalies (tetralogy of Fallot, heart tumor, fetal small bowel dilatation, and pulmonary sequestration). In addition, one newborn of a mother infected within the Delta variant wave period and one newborn of a mother with an unknown medical history were excluded. Of the total newborns, 60 were boys and 55 were girls. There were 16 newborns of mothers who had a history of Omicron 2019-nCoV infection and 99 babies were born to mothers who did not. All mothers were infected during the 3rd trimester. Of the mothers infected with Omicron 2019-nCoV, 7 newborns were of symptomatic mothers and 4 were of isolated mothers (Fig. 1). The mean days from the confirmed date of infection to delivery were 25.36 ± 19.47 d. The maximum number of days from the confirmed date of infection to delivery was 64, while the median was 18 d. In Symptomatic mothers, the mean days form infection to delivery were 7.14 ± 5.87 d. And maximum number of days from the confirmed date of infection to delivery was 14 d, while the median was 6 d.

There were no significant differences in IUP at childbirth, maternal age, CS ratio, male-to-female ratio, and pregnancy complications such as PROM, G-HTN, and GDM in the presence or absence of COVID history (*p* > 0.05). Moreover, there was no significant difference in A/S, SGA ratio, LGA ratio, and twin ratio (*p* > 0.05).

**Fig. 2:**
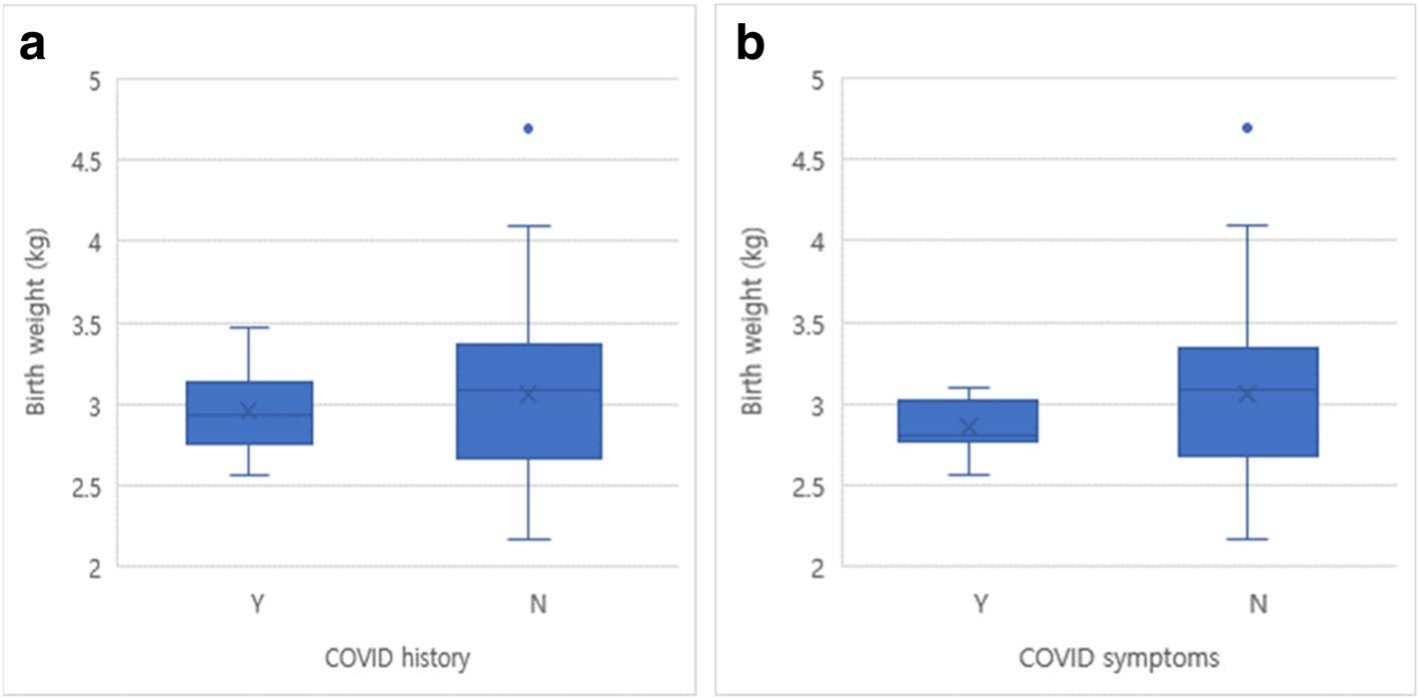
Birth weight by COVID history and COVID symptoms. **a** Birth weight by COVID history *; **b** Birth weight by COVID symptoms ***p value = 0.049 **p value = 0.041

The average birth weight of newborns of mothers with a COVID-19 history was 2.958 ± 0.272 kg, whereas that of newborns of mothers without a COVID-19 history was 3.064 ± 0.461 kg, which was statistically significant (*p* = 0.049) (Fig. 2). However, there was no significant difference (*p* > 0.05) in the number of patients who underwent NRP, required respiratory support after 12 h of birth, or had a suspected septic condition. No mortalities were reported in either group. A total of 13 babies underwent the 2019-nCoV PCR test, and all the tests were reportedly negative on 2019-nCoV PCR performed at 24 and 48 h after birth (Table 1). All of the babies with respiratory problems were well-treated and discharged within 14 days of their admission.

**Table 1:** Perinatal outcomes by Omicron COVID history

	**COVID History**		
	Y	N	p
No	16	99	
No. of 2019-nCoV test done (positive)	7 (0)	6 (0)	
IUP (weeks)	37.75 ± 0.931	37.86 ± 0.869	0.574
Maternal age (years)	33.25 ± 4.973	33.05 ± 4.263	0.923
A/S			
1 AS	8.44 ± 0.964	8.57 ± 0.949	0.413
5 AS	9.56 ± 0.629	9.78 ± 0.464	0.117
M: F (n: n)	8: 8	52: 47	0.530
Birth weight (kg)	2.958 ± 0.272	3.064 ± 0.461	0.049
No. (%)			
SGA	6 (37.50)	29 (29.29)	0.330
AGA	10 (62.50)	64 (64.65)	
LGA	0 (0.00)	6 (6.06)	
Place of delivery, No. (%)			
Inborn	13 (81.25)	93 (93.94)	0.110
Outborn	3 (18.75)	6 (6.06)	
Delivery mode, No. (%)			
SVD	4 (25.00)	17 (17.17)	0.488
CS	12 (75.00)	82 (82.83)	
Multiple delivery, No. (%)	2 (12.50)	22 (22.22)	0.517
NRP done, No. (%)	5 (31.25)	24 (24.24)	0.549
PEEP	4 (25.00)	19 (19.19)	
PPV	1 (6.25)	5 (5.05)	
Resp support after 12 h, No. (%)	4 (25.00)	15 (15.15)	0.300
TTN	4 (25.00)	13 (13.13)	1.000
RDS	0 (0.00)	2 (2.02)	1.000
Air leak	1 (6.25)	3 (3.03)	0.455
Suspicious sepsis, No. (%)	2 (12.50)	7 (7.07)	0.610
Complication of pregnancy, No. (%)			
PROM	0 (0.00)	1 (1.01)	
G-HTN, PE	0 (0.00)	7 (7.07)	
DM, GDM	3 (18.75)	12 (12.12)	

*COVID-19* Coronavirus disease, *2019-nCoV* Novel coronavirus, *IUP* Intrauterine pregnancy weeks, *A/S* Apgar score, *M* Male, *F* Female, *SGA* Small-for-gestational-age, *AGA* Average-for-gestational-age, *LGA* Large-for-gestational-age, *NRP* Neonatal resuscitation program, *PEEP* Positive end-expiratory pressure, *PPV* Positive pressure ventilation, *TTN* Transient tachypnea of the newborn, *RDS* Respiratory distress syndrome of newborn, *PROM* Prolonged rupture of membranes, *G-HTN* Gestational hypertension, *PE* Pre-eclampsia, *DM* Diabetes mellitus, *GDM* Gestational diabetes mellitus

When comparing mothers with symptoms of upper respiratory tract infection and mothers without symptoms, the IUPs at childbirth, maternal age, the CS ratio, and the male-to-female ratio had no statistically significant difference (*p* > 0.05).

However, A/S at 1 and 5 min were respectively 8.00 ± 1.000 and 9.29 ± 0.756 for mothers with upper respiratory tract infection symptoms, and 8.58 ± 0.939 and 9.78 ± 0.460 for asymptomatic mothers. This demonstrated a statistically significant difference at 5 min (*p* = 0.019). The mean birth weights of children born to mothers with symptoms of upper respiratory tract infection and asymptomatic mothers was 2.857 ± 0.183 kg and 3.062 ± 0.450 kg, respectively, which was statistically significant (*p* = 0.041) (Fig. 2). The ratio of SGA, LGA, and the ratio of twins did not demonstrate any significant results. (*p* > 0.05). The following were not significant between the two groups: performance of NRP, the requirement for respiratory support until 12 h after birth, and suspected sepsis (*p* > 0.05) (Table 2).

**Table 2:** Perinatal outcomes by Omicron COVID Symptoms and Omicron quarantine by Korea

	**COVID Symptoms**			**COVID quarantine**		
	Y	N	p	Y	N	p
No	7	108		4	111	
IUP (weeks)	38.43 ± 0.976	37.81 ± 0.859	0.068	38.00 ± 1.155	37.84 ± 0.869	0.768
A/S						
1 AS	8.00 ± 1.000	8.58 ± 0.939	0.098	7.25 ± 0.500	8.59 ± 0.928	0.413
5 AS	9.29 ± 0.756	9.78 ± 0.460	0.019	8.75 ± 0.500	9.78 ± 0.455	0.117
M: F (n: n)	2: 5	58: 50	0.257	1: 3	59: 52	0.348
Birth weight (kg)	2.857 ± 0.183	3.062 ± 0.450	0.041	2.845 ± 0.236	3.057 ± 0.448	0.215
Delivery Mode, No. (%)						
SVD	2 (28.57)	19 (17.59)	0.610	1 (25.00)	20 (18.02)	0.559
CS	5 (71.43)	89 (82.41)		3 (75.00)	91 (81.98)	
NRP done, No. (%)	2 (28.57)	27 (25.00)	1.000	2 (50.00)	27 (24.32)	0.264
PEEP	2 (28.57)	21 (19.44)		2 (50.00)	21 (18.92)	
PPV	0 (0.00)	6 (5.55)		0 (0.00)	6 (5.41)	
Resp support after 12 h, No. (%)	3 (42.86)	16 (14.81)	0.087	3 (75.00)	16 (14.41)	0.014*
TTN	3 (42.86)	14 (12.96)	0.065	3 (75.00)	14 (12.61)	0.010**
RDS	0 (0.00)	2 (1.85)	1.000	0 (0.00)	2 (19.80)	1.000
Air leak	1 (14.29)	3 (2.78)	0.225	1 (25.00)	3 (2.70)	0.134

*COVID-19* Coronavirus disease, *IUP* Intrauterine pregnancy weeks, *A/S* Apgar score, *M* Male, *F* Female, *SVD* Spontaneous vaginal delivery, *CS* Caesarian section, *NRP* Neonatal resuscitation program, *PEEP* Positive end-expiratory pressure, *PPV* Positive pressure ventilation, *TTN* Transient tachypnea of the newborn, *RDS* Respiratory distress syndrome of newborn

^*^OR = 10.275

^**^OR = 11.929

Lastly, when compared with isolated participants, other values did not demonstrate a significant difference; however, patients requiring respiratory support 12 h after birth demonstrated a significant difference (OR, 10.275) The presence or absence of TTN was significant (OR 11.929; *p* = 0.014; *p* = 0.01) (Table 2).

## Discussion

In a single center-based retrospective study, maternal COVID was significantly associated with respiratory distress and less birth weight in full-term neonates.

In previous studies, there have been various views regarding the complications that can occur in newborns of COVID-19-positive mothers [4–8]. According to Nayak et al., hypoxic ischemic encephalopathy, meconium-stained amniotic fluid, and CS increased, but other results showed no significant differences [6]. According to Norman et al., morbidities such as RDS, TTN, and hyperbilirubinemia increased in the presence of 2019-nCoV [9]. In addition, Wróblewska–Seniuk et al. reported an increase in RDS and TTN rates [10].

Expressed viral RNA of 2019-nCoV has been discovered in the placenta and fetal membranes of mothers infected with COVID-19 [16]. However, it is known that transplacental transmission of 2019-nCoV to newborns is rare [17]. Studies have shown that CS delivery is preferred by mothers infected with COVID-19 to prevent infection through airborne transmission to the newborn during childbirth. This may result in respiratory complications [4, 6], as maternal CS is a major risk factor for TTN and RDS [18].

In this study, the proportion of CS was high. According to data released in 2018, the Korean CS rate and Organization for Economic Co-operation and Development (OECD) average were 42.3% and 21%, respectively [12]. Various factors are listed as the possible causes. However, the gestational age at childbirth is reportedly increasing compared to that in the OECD. The high CS rate in this study can be attributed to the study site being a single-center tertiary hospital. In this study, the total CS rate was 81.74%, and it was 79.96% in the previous 3 years. Furthermore, the high CS rate was not attributed to COVID-19 because the proportion of CS did not increase even when COVID-19 was present.

In addition, we reported that the birth weight was significantly < 2500 g for full-term newborns of mothers with a history of COVID-19 or who were symptomatic. There was no significant difference in the ratio of SGA and LGA. In this study, all mothers were infected with COVID-19 during the 3rd trimester, near full-term. A widely known symptom of COVID is taste impairment [19], and taste impairment leads to low calorie intake [20]. Third trimester fetal growth retardation is influenced by poor maternal weight gain [21]. But a small number of calories has minimal effects on fetal growth [22]. In this study, there were no placental anomalies in mothers with a history of COVID-19, and no patients with G-HTN. We can infer that the 3rd trimester was affected by maternal COVID-19 symptoms rather than by COVID-19 itself, but there were no significant differences between self-isolated participants and those who were unrestricted. Further, the lower birthweight of newborns was attributed to COVID-19 symptoms.

The 5-min A/S was significantly lower in full-term babies born to symptomatic mothers, and as in a previous study, TTN rates significantly increased in self-isolated participants and some required ventilatory support [9, 10]. It is thought that, like other variants, the Omicron variant 2019-nCoV increases respiratory morbidity. However, its respiratory effects are amplified closer to the date of birth of a full-term infant. Although the exact cause is unknown, respiratory conditions such as COVID-19 may be linked to TTN, just as maternal asthma is linked to TTN [23].

Unlike previous studies, this study was based on the Omicron variant 2019-nCoV and its effects on newborns were analyzed. The currently dominant Omicron variant 2019-nCoV has low pathogenicity but high transmissibility, unlike other variants. This has resulted in a global increase in the incidence of COVID-19 [1] and the adoption of social distancing practices [1, 24].

This study was limited by the single-center base, resulting in a small number of participants. Therefore, additional multi-centered studies with an increased number of participants are required. Further, unvaccinated mothers and those who contracted 2019-nCoV in their 3rd trimester were enrolled in this study. A future analysis of 2019-nCoV infection by gestational weeks may be beneficial.

## Data Availability

The datasets generated and/or analyzed during the current study are not publicly available [REASON WHY DATA ARE NOT PUBLIC] but are available from the corresponding author on reasonable request.

## Abbreviations

COVID-19: Coronavirus disease
2019-nCoV: Novel coronavirus
Ct: Cycle threshold
RdRp: RNA-dependent RNA polymerase
E: Envelope gene
PCR: Polymerase chain reaction
G-HTN: Pre-eclampsia
PROM: Prolonged rupture of membranes
GDM: Gestational diabetes mellitus
VD: Vaginal delivery
CS: Cesarean section
IUP: Intrauterine pregnancy weeks
A/S: Apgar score
NRP: Neonatal resuscitation program
SGA: Small-for-gestational-age
AGA: Average-for-gestational-age
LGA: Large-for-gestational-age
TTN: Transient tachypnea of the newborn
RDS: Respiratory distress syndrome of newborn
OECD: Organisation for Economic Co-operation and Development
